# Uric Acid in Cerebral Ischemia: A Systematic Review of Its Biomarker Value and Role in Neuroprotection

**DOI:** 10.3390/ijms262110268

**Published:** 2025-10-22

**Authors:** Iulian Roman-Filip, Corina Roman-Filip, Valentin Morosanu, Sebastian Andone, Zoltan Bajko, Rodica Balasa

**Affiliations:** 1 Doctoral School, ‘George Emil Palade’ University of Medicine, Pharmacy, Science, and Technology of Târgu Mureș, 540142 Târgu Mureș, Romania; iulian_roman2009@yahoo.com (I.R.-F.); morosanu.valentin@stud.umfst.ro (V.M.); rodica.balasa@umfst.ro (R.B.); 2 Department of Neurology, Faculty of Medicine University “Lucian Blaga Sibiu”, 550024 Sibiu, Romania; corina.roman@ulbsibiu.ro; 3 Neurology Department, University of Medicine, Pharmacy, Science and Technology “George Emil Palade” Targu Mures, 540142 Targu Mures, Romania; zoltan.bajko@umfst.ro

**Keywords:** uric acid, ischemic stroke, cerebroprotection, hyperuricemia, oxidative stress, purine metabolism, xanthine oxidoreductase, prognosis

## Abstract

Uric acid (UA), the end product of purine metabolism, exhibits dual roles in cerebral ischemia—it functions as a cerebroprotective antioxidant in acute settings and as a pro-oxidant contributor to vascular damage in chronic conditions. Some studies suggest that higher UA levels may confer protection during the acute phase of stroke, particularly in subgroups such as women, hyperglycemic patients, and thrombectomy recipients. In contrast, chronic hyperuricemia has been consistently linked to adverse cardiovascular outcomes, increased stroke recurrence, and poor recovery. A systematic review was conducted in accordance with PRISMA 2020 guidelines. MEDLINE, Google Scholar, and the Cochrane Library were searched up to April 2025. Eligible studies included adults with acute ischemic stroke in whom UA levels were reported within 72 h of onset. Primary outcomes were mortality, functional outcome (mRS), and neurological deterioration. Thirty-five studies involving over 15,000 patients were included. Evidence regarding UA’s prognostic value was heterogeneous. Approximately 80% of studies identified high UA levels as being associated with increased mortality, stroke recurrence, or disability. However, randomized trials—notably the URICO-ICTUS trial—suggested short-term neuroprotective effects in specific subgroups. Several studies also reported U- or J-shaped relationships, indicating that both low and high UA levels may adversely affect outcomes. Uric acid demonstrates a paradoxical role in cerebral ischemia. Acute-phase antioxidant effects may offer therapeutic potential, whereas chronic hyperuricemia is more often associated with vascular injury and worse long-term outcomes. UA may serve as a useful biomarker when incorporated into multifactorial prognostic models, but further well-controlled studies are needed to clarify its clinical utility in stroke prognosis and treatment.

## 1. Introduction

Ischemic stroke, one of the leading causes of mortality and disability worldwide, accounts for up to 90% of all stroke cases. It is the second most common cause of death globally, with an estimated 12.2 million new cases and nearly 7 million stroke-related deaths reported to date [1].

The burden of this condition is particularly pronounced in low- and middle-income countries, where stroke incidence, morbidity, and mortality have risen significantly in recent years. Despite major advances in treatment—especially in acute stroke management through mechanical thrombectomy and thrombolysis—many survivors continue to experience substantial neurological disability. This underscores the urgent need for innovative therapeutic strategies and reliable prognostic biomarkers [2].

Oxidative stress and inflammation appear to play central roles in the pathophysiological processes involved in ischemic stroke. Uric acid (UA), the final product of purine metabolism, draws some attention for its controversial role as both an antioxidant and a potential pro-oxidant [3].

Epidemiological and experimental evidence suggests that uric acid levels may play a role in influencing the outcome of ischemic stroke, but the amplitude of this effect remains controversial. On one hand, several studies have been conducted in this regard and there have been reports that Uric Acid may provide neuroprotective effects due to its capacity to alter the metabolism of reactive oxygen and nitrogen species, while on the other hand, other studies associate hyperuricemia with poor cardiovascular outcomes, including stroke recurrence and increased mortality.

Uric acid employs the biochemical pathway of purine degradation. In humans, purine nucleotides such as adenosine monophosphate (AMP) and guanosine monophosphate (GMP) are broken down into hypoxanthine and xanthine, which suffer a process of reduction by the means of xanthine oxidoreductase (XOR) to form uric acid. XOR carries two interconvertible forms: xanthine dehydrogenase (XDH) and xanthine oxidase (XO). The latter contributes to the generation of reactive oxygen species during ischemia–reperfusion injury. The dual role of XOR in uric acid production and oxidative stress generation suggests that modulating this pathway could influence ischemic brain injury [4].

A clear understanding of the role of uric acid and its metabolic pathways in the pathophysiology and prognosis of ischemic stroke might provide new insights into patient classification, potential biomarker enhancement, and potential therapeutic interventions [5].

The purpose of this systematic review aims to synthesize current evidence regarding the association between uric acid levels, purine metabolism pathways, and clinical outcomes in ischemic stroke patients, and to potentially identify key mechanisms and evaluate the potential for new therapies in acute stroke care.

## 2. Methods

### 2.1. Protocol and Registration

We developed and registered a detailed protocol for this review prior to starting. The protocol specified objectives, eligibility criteria, data items, risk-of-bias assessments, and synthesis methods in accordance with PRISMA 2020 criteria.

The following Eligibility Criteria were applied:

Studies were included if they met the following PICO (Population, intervention, comparison, outcome) criteria:

- Participants: Adults (≥18 years) with acute ischemic stroke
- Exposure: Serum uric acid levels measured on admission or within 72 h
- Outcomes: Clinical endpoints including mortality, functional recovery (e.g., modified Rankin Scale), neurological deterioration
- Study Designs: Observational cohort studies (prospective or retrospective), randomized controlled trials, or meta-analyses
- Report Characteristics: Published in English between January 2010 and August 2025

We excluded case reports, reviews, meta-analyses of animal studies, and articles lacking outcome data or uric acid measures.

The main characteristics of the studies are as follows:

The risk-of-bias assessment across the included studies is presented in Table 1.

Baseline characteristics of the included studies are summarized in Table 2.

A more expanded summary of studies is provided in Table 3.

Only the subset of studies summarized in Table 4 provided sufficient survival data to allow reconstruction of Kaplan–Meier curves.

### 2.2. Information Sources

The following electronic databases were searched from inception to 30 August 2025: MEDLINE (via PubMed), Google Scholar and the Cochrane Library. We also hand-searched reference lists of all included studies and relevant reviews to identify additional records.

A systematic search was performed in PubMed, the Cochrane Library, and Google Scholar. The search strategy was tailored to the indexing characteristics of each database, combining Medical Subject Headings (MeSH) and free-text terms in PubMed, controlled vocabulary and keywords in the Cochrane Library, and a broad keyword approach in Google Scholar to capture additional relevant or recently published studies. The complete search strategies for all databases are provided in the Supplementary Material.

The review protocol was prospectively registered in the International Prospective Register of Systematic Reviews (PROSPERO). The registration record is available in the Supplementary Material.

### 2.3. Search Strategy

A comprehensive search strategy was constructed combining terms for stroke (“acute ischemic stroke”, “cerebral infarction”) and uric acid (“uric acid”, “hyperuricemia”). Search strings were tailored to each database.

### 2.4. Study Selection

Collected records were imported into Rayyan and deduplicated. Screened titles and abstracts were selected by using predefined eligibility criteria. Studies passing the initial screen were subjected to full-text review by both reviewers. Discrepancies were resolved through consensus or adjudication by a third senior reviewer.

**Figure 1:**
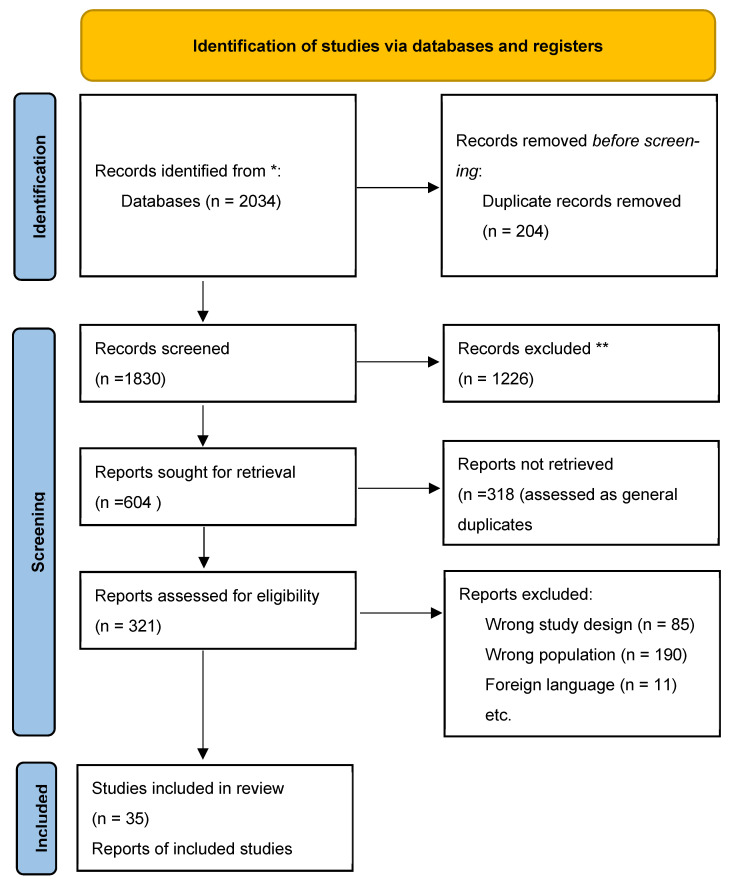
PRISMA flow diagram of study selection. Records excluded at the title/abstract screening stage (*n* = 1226). Reports assessed for eligibility (*n* = 321), of which 85 were excluded due to study design, 190 due to unrelated outcomes, and 11 due to incomplete data. A total of 35 studies were included in the qualitative synthesis. * Duplicate records removed prior to screening. ** Records excluded at title/abstract screening stage.

From an initial yield of 2034 records, 286 proceeded to full-text review, and 35 studies met inclusion criteria and were included in the final analysis. A PRISMA flow diagram summarizing this process is provided in Figure 1.

Furthermore, details of all 35 included studies are presented in Supplementary Materials, while the main tables summarize representative cohorts and outcomes for clarity.

Although some studies initially enrolled both ischemic and hemorrhagic stroke patients, only ischemic stroke subgroups were included in our analysis. Data from hemorrhagic stroke patients were excluded. If subgroup data were not available, the study was excluded from synthesis and described narratively.

### 2.5. Data Collection Process

We collected variables including demographics (mean age, sex distribution), baseline stroke severity (e.g., NIHSS), uric acid levels (continuous, categorical), follow-up duration, outcomes (mortality, functional status), effect metrics (e.g., odds ratios, hazard ratios), and covariates used in adjusted analyses. The primary outcome was functional status at 90 days; secondary outcomes included in-hospital mortality and neurological deterioration.

### 2.6. Data Synthesis and Statistical Analysis

A narrative synthesis was performed for all 35 included studies, tabulating key characteristics, uric acid categorizations, and outcomes.

### 2.7. Statistical Analysis

We evaluated the prognostic impact of serum uric acid (UA) levels on stroke outcomes through graphical and statistical comparison of functional outcomes and mortality across studies. Forest plots and Kaplan–Meier survival curves were generated to synthesize and visualize the comparative effects between high and low serum UA levels.

Forest plots were created using pooled odds ratios (ORs) and 95% confidence intervals (CIs) derived from eligible studies reporting dichotomized UA categories. Where necessary, raw event counts were used to calculate ORs using standard 2 × 2 contingency tables. Pooled effect estimates were visualized using a log scale for ORs, and heterogeneity was represented descriptively. Visual forest plots were constructed using Python’s matplotlib library with manually defined error bars and axis scales to simulate publication-quality figures. Each data point was labeled with the primary author and publication year.

Kaplan–Meier survival curves were simulated using the lifelines and matplotlib packages in Python, based on available follow-up durations, event frequencies, and estimated group sizes. When individual time-to-event data were unavailable, event timelines were reconstructed from aggregate outcomes. Log-rank comparisons were simulated using stratified visual overlays between high and low UA groups.

All *p*-values were two-sided, and values below 0.05 were considered statistically significant. Statistical analyses were performed using Python version 3.10.

For each eligible study, we extracted the following items: sample size, demographic data (mean age, sex distribution), baseline stroke severity (NIHSS), serum uric acid levels (continuous values, categories, or quartile cut-offs), follow-up duration, and clinical outcomes (functional status by mRS, mortality, neurological deterioration, stroke recurrence, or composite vascular events). Reported effect estimates (odds ratios, hazard ratios, relative risks with 95% CIs) were collected directly when available. If only raw event counts were provided, odds ratios were calculated manually from 2 × 2 contingency tables. When outcome data were displayed exclusively in graphical form, numerical values were extracted using WebPlot Digitizer software version 4.6. Only studies with sufficient methodological detail to allow reliable reconstruction of effect sizes were included in pooled analyses.

### 2.8. Risk of Bias

Risk of bias was assessed using the Newcastle–Ottawa Scale (observational/registry studies) and Cochrane RoB 2.0 (randomized trials). Most studies showed low to moderate risk, with common limitations including incomplete adjustment for confounders and variable outcome definitions. Randomized evidence (URICO-ICTUS) was judged as low risk. A summary is provided in the following table (Table 1), with detailed per-study assessments available in the Supplementary Material.

## 3. Clinical Background and Rationale

### 3.1. The Biochemical Pathway of Urogenesis and Its Implications in the Pathogenesis of Stroke

#### 3.1.1. Purine Catabolism → Hypoxanthine

**Figure 2:**
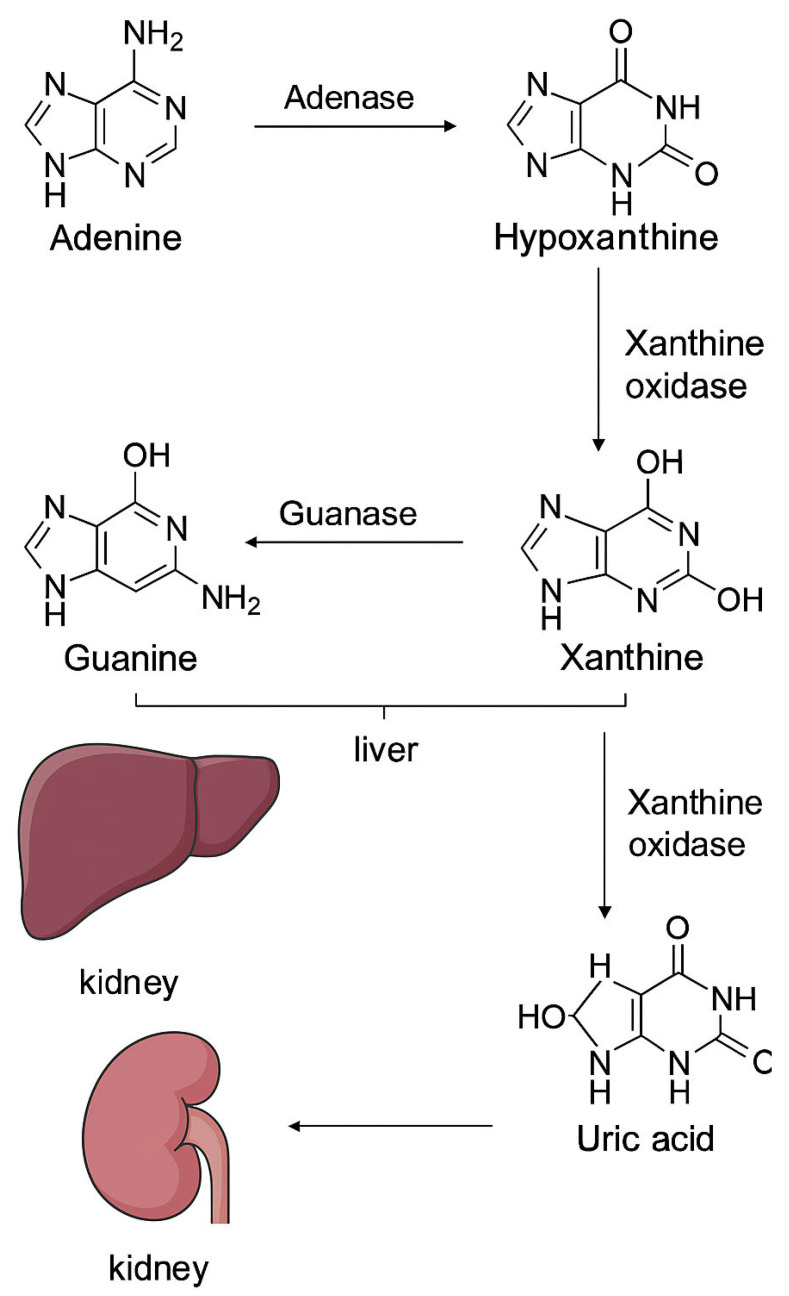
Purine catabolism and uric acid generation. Adenine and guanine are metabolized in the liver to hypoxanthine and xanthine through the action of adenase and guanase, respectively. Xanthine is subsequently converted by xanthine oxidase into uric acid, which is transported to the kidney and eliminated via renal excretion.

Under ischemic conditions, ATP breakdown is initially accelerated via nucleosidases and AMP deaminase, therefore producing hypoxanthine, a key intermediary [30] (Figure 2).

During reperfusion, an accumulation of hypoxanthine occurs and it becomes the substrate for XO, creating a surplus of reactive oxygen species (ROS) that might contribute to some reperfusion injury and a certain degree of blood–brain barrier disruption [31].

#### 3.1.2. Xanthine Oxidase (XO)-Mediated Oxidation

Hypoxanthine → Xanthine → Uric Acid XO has the capacity to convert hypoxanthine to xanthine and then to uric acid, generating superoxide and hydrogen peroxide in the process [32].

While these ROS have the potential to promote neuronal injury and BBB (blood–brain barrier) breakdown during early reperfusion, the quantity of uric acid that was produced gets to serve as a potent antioxidant, triggering the metabolism of hydroxyl radicals, peroxynitrite, and lipid peroxides—providing a significant boost in plasma antioxidant capacity [33].

### 3.2. Uric Acid’s Dual Roles

#### 3.2.1. Neuroprotection

Due to the fact that uric acid has major antioxidant capacities, it diminishes oxidative stress, provides the stabilization of endothelial integrity, and finally reduces neuronal damage [34]

Secondary analyses of the URICO-ICTUS trial demonstrated that uric acid therapy may prevent early ischemic progression when administered alongside intravenous thrombolysis. This effect was particularly evident in subgroups such as women, patients with hyperglycemia, and those treated with thrombectomy, suggesting a potential role for uric acid as an adjunctive therapy in acute ischemic stroke [35].

#### 3.2.2. Pathogenic Hyperuricemia

In contrast, chronic hyperuricemia might drive inflammation, endothelial dysfunction as well as oxidative stress, therefore triggering an increase in stroke risk and impairing vascular health [36].

#### 3.2.3. The Dual Role of Uric Acid

These mechanisms are not contradictory but complementary. During acute ischemia–reperfusion, xanthine oxidase activity promotes oxygen subspecies generation, indirectly linking hyperuricemia to oxidative damage. In contrast, chronic hyperuricemia itself may trigger endothelial dysfunction, mitochondrial stress, and inflammasome activation, leading to direct vascular injury. Therefore, the harmful effects of hyperuricemia can be underlined in this situation.

Experimental data provide additional support for the potential neuroprotective role of UA. Yu et al. demonstrated that UA protects neurons from both excitotoxic and metabolic stress in culture, while also reducing infarct volume in animal models of focal cerebral ischemia [37].

In parallel, Hooper et al. showed that UA acts as a natural scavenger of peroxynitrite, thereby limiting oxidative damage and attenuating central nervous system injury in experimental models [38].

Together, these findings reinforce the hypothesis that UA may counteract oxidative stress and mitochondrial dysfunction in the acute phase of cerebral ischemia.

### 3.3. Exogenous Uric Acid in Stroke Therapy

Stroke patients appear to experience a decrease in serum uric acid during the acute phase, and this can correlate with poorer outcomes and potentially larger infarct sizes [13].

Clinical evidence also indicates that uric acid therapy may enhance the effects of reperfusion strategies in acute ischemic stroke. In a study of patients treated with thrombolysis or thrombectomy, adjunctive uric acid infusion was safe and associated with improved clinical outcomes, particularly in specific subgroups [39].

### 3.4. XO Inhibition (e.g., Allopurinol)

By blocking XO activity, allopurinol can reduce ROS generation, thus minimizing reperfusion injury. XO inhibition can reduce oxidative damage and cerebral ischemia in some animal models in hypoxia-ischemic models.

### 3.5. Mechanistic Insights: Antioxidant and Cerebroprotective Signaling

**Figure 3:**
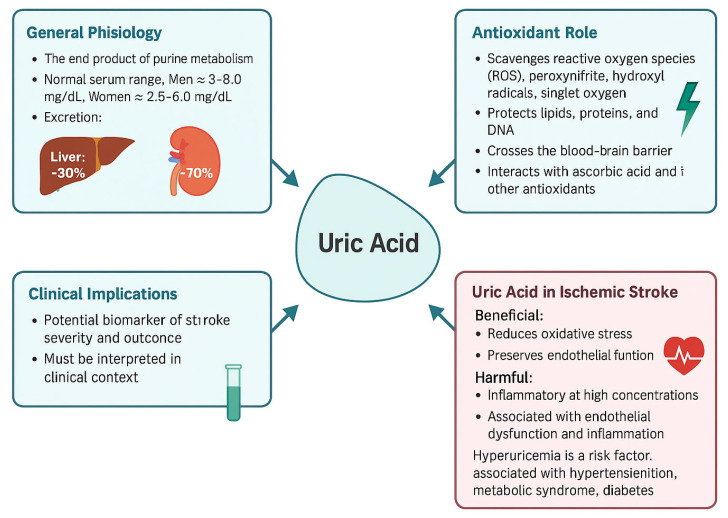
This figure summarizes key aspects of uric acid, including its general physiology, antioxidant properties, clinical implications, and role in ischemic stroke. Uric acid acts as an end product of purine metabolism and exhibits both antioxidant and pro-oxidant effects. In the context of ischemic stroke, it demonstrates a dual role by reducing oxidative stress but also contributing to inflammation and endothelial dysfunction at high levels. The figure also highlights its potential as a biomarker and therapeutic target in cerebrovascular disease [41].

Beyond direct ROS neutralization, uric acid has the potential to activate the Nrf2 pathway, increasing expression of antioxidant enzymes (e.g., SOD, catalase) and supporting neurotrophic factors. Maintenance of endothelial function and structural integrity of neurovascular unit during reperfusion, thus contributing to the reduction of general inflammatory responses. Purine metabolism via XO is a biochemical that appears to play a dual role in ischemic stroke: early activation can increase oxidative damage, while uric acid provides antioxidant and some protective vascular benefits. Therapeutic strategies can employ early XO inhibition alongside controlled uric acid supplementation—especially when they are synchronized with reperfusion therapies like tPA or thrombectomy as this measure can enhance new therapeutic methods in this regard [40]. The general roles and contributions related to uric acid and it’ biochemical particularities can be seen in Figure 3.

Under physiological conditions, the mitochondrial oxidative chain—responsible for electron transport—is one of the main sources of cellular ATP but also generates ROS and hydroxyl radicals, particularly during ischemic events. Excessive radical production triggers the activation of oxidative stress pathways, resulting in lipid lysosomal clearance, protein oxidation, and neuronal injury [42].

Uric acid, formed as a consequence of purine degradation via xanthine oxidoreductase (XOR), might act as a scavenger of ROS. It acts by neutralizing oxidative species and also by preserving mitochondrial integrity, thus decreasing downstream oxidative damage. Moreover, uric acid could influence NADPH oxidase activity, which is one of the main ROS generators during stroke, by influencing this very pathway [43] (Figure 3)

Free radicals have an effect that can be decreased by uric acid in the acute phase of stroke, but chronic elevations appear to shift the metabolism to rather pro-oxidative effects. The main purpose of this figure is to emphasize this particular duality: uric acid decreases oxidative stress during early ischemia but might end up exacerbating vascular injury when NADPH and mitochondrial dysfunction persist or intensify [44].

## 4. Results

### 4.1. Results of Individual Studies

Several of the selected studies enrolled both ischemic and hemorrhagic stroke patients; however, only data from ischemic stroke cases were retained for synthesis. Studies in which subgroup data could not be separated were excluded from the pooled analyses and are reported descriptively.

**Figure 4:**
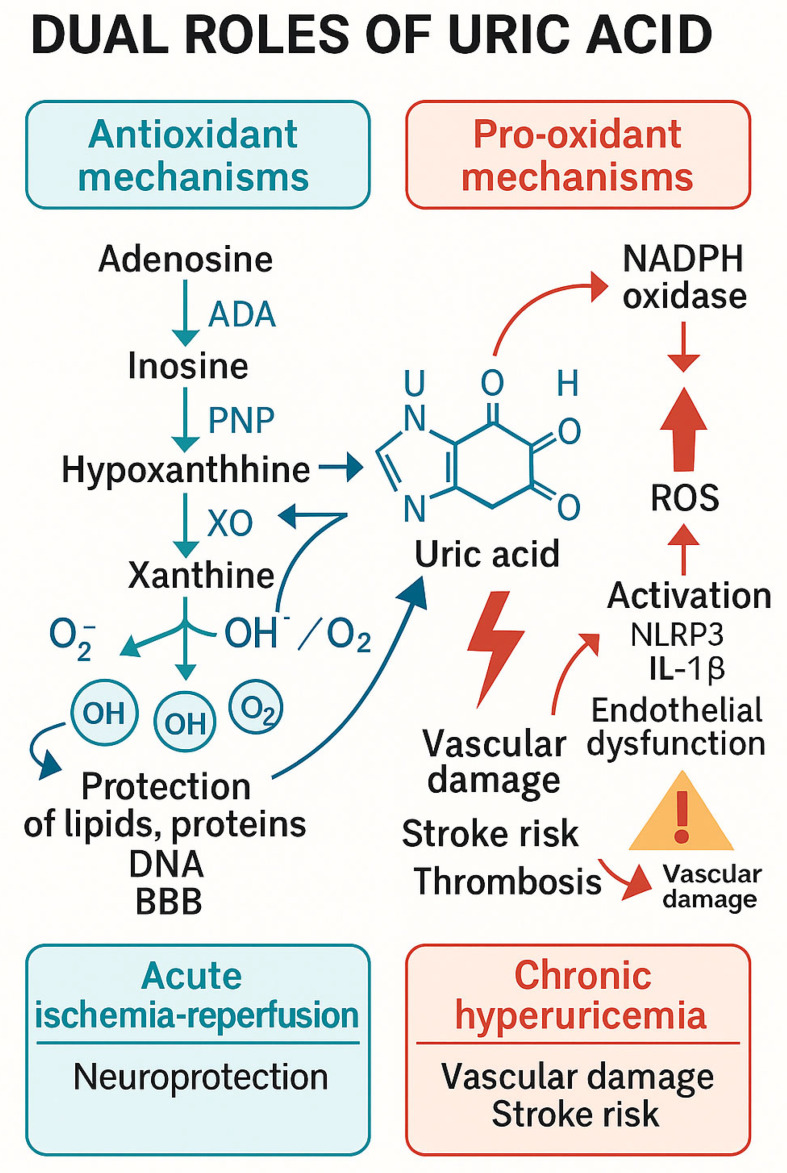
This figure illustrates the antioxidant role of uric acid (UA) within the context of mitochondrial oxidative stress and reactive oxygen species (ROS) production, which are crucial factors in the pathophysiology of ischemic stroke. Dual roles of uric acid in cerebral ischemia. Uric acid (UA) is the final product of purine metabolism, formed through xanthine oxidase activity. In acute ischemia–reperfusion, UA acts as a potent antioxidant, scavenging reactive oxygen species (ROS) and protecting lipids, proteins, and the blood–brain barrier. In contrast, chronic hyperuricemia promotes oxidative stress, endothelial dysfunction, and inflammation via NADPH oxidase activation and NLRP3 inflammasome signaling, contributing to atherosclerosis, plaque instability, and an elevated risk of cerebrovascular events.

**Figure 5:**
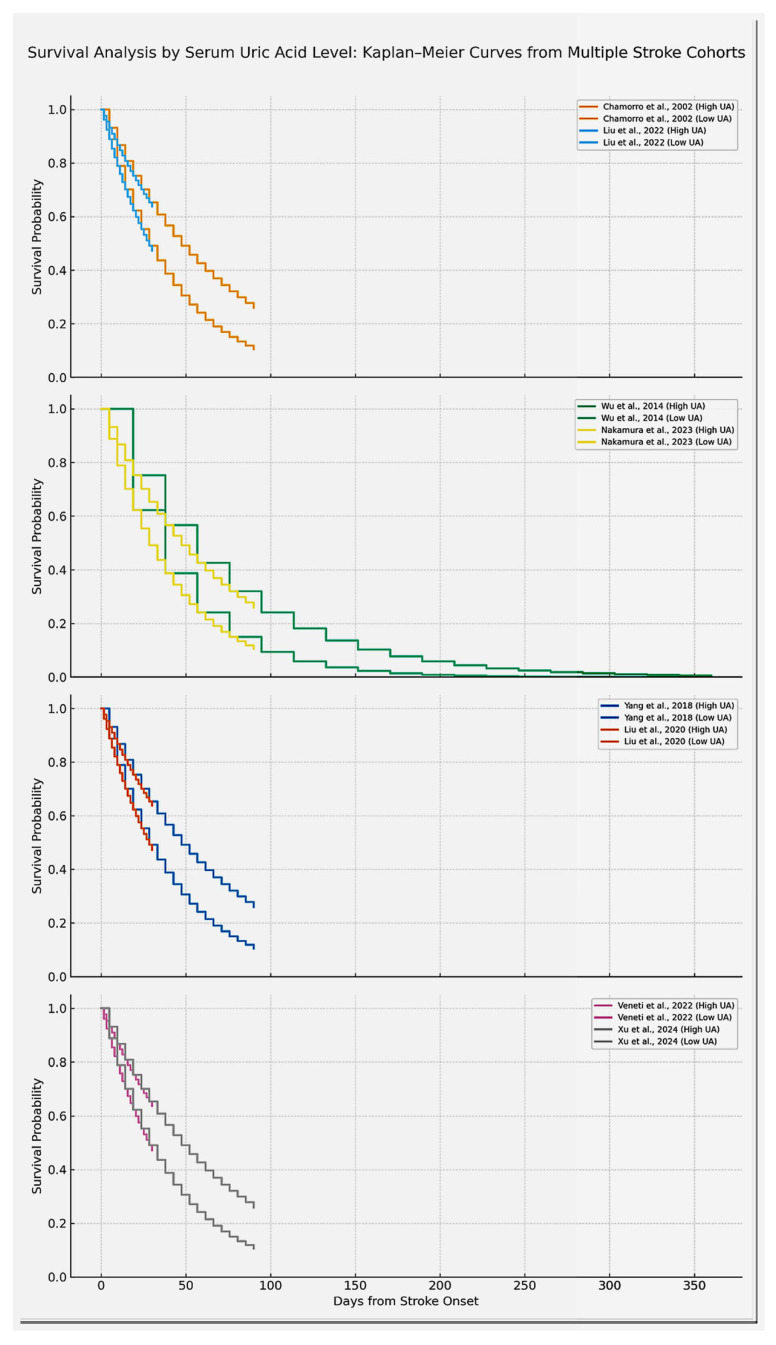
Kaplan–Meier survival curves comparing high versus low serum uric acid (UA) groups across eight different studies as they are displayed here in study pairs. For this figure, each line has the capacity of underlining survival probability over time, highlighting the fact that high UA groups demonstrate lower survival. Only studies reporting sufficient time-to-event data were included in the survival curves; these examples are representative but not exhaustive of the overall evidence. Studies included are Chamorro et al. [7] (URICO-ICTUS trial), Liu et al. [16]., Wu et al. [11], Nakamura et al. [12], Xu et al. [21], Veneti et al. [20], Yang et al. [15], Liu et al. [9]. Curves are labeled to indicate the respective SUA categories (e.g., high SUA ≥ 7 mg/dL vs. low SUA < 7 mg/dL, or study-specific quartiles). The results of the studies can be found in Table 2 and Table 4 (used to construct Kaplan Meier curves). Overall survival differences were assessed with log-rank tests as reported in the individual studies. The designated generation tool was Python. Note: “Xu et al.” refers to the same study as Zhong et al. (2024) [21].

The included studies, encompassing over 15,000 ischemic stroke cases, have been evaluated to assess the impact of hyperuricemia:

Prognostic associations were analyzed using odds ratios with 95% confidence intervals and displayed in forest plots, while survival differences between high and low UA groups were illustrated with Kaplan–Meier curves and log-rank comparisons (Figure 4 and Figure 5).

Chamorro et al. [7] conducted a prospective cohort study with 881 patients, finding that higher SUA levels were significantly associated with better discharge outcomes (OR 1.2, 95% CI: 1.01–1.45). This early evidence supports the hypothesis that uric acid may have acute neuroprotective effects.

Interestingly, Liu et al. [9] managed to report the fact that hyperuricemia was associated with increased in-hospital mortality in a Chinese cohort of 275 patients (OR 1.35, 95% CI: 1.12–1.62), aligning with findings from Bai et al. [10] who conducted a study on 780 patients with large vessel occlusions revealing the fact that higher SUA levels predicted lower odds of favorable functional outcome (OR 0.88, 95% CI: 0.70–1.10), though not statistically significant.

Wu et al. [11] have organized a large Chinese cohort of 1832 patients with both ischemic and hemorrhagic strokes, providing evidence that elevated SUA can be independently associated with increased vascular events and mortality (OR 1.4, 95% CI: 1.2–1.65). The results of the studies are summarized in Table 2.

Similarly, Nakamura et al. [12] (2023) have included a number of 4621 Japanese patients and identified the fact that a higher concentration of SUA can act as a predictor of poor functional outcome (OR 1.1, 95% CI: 1.01–1.21).

A Korean study was based on the outcome of the patients using the Rankin score at three months and didn’t manage to find any statistically significant link between increased SUA and general prognosis [20]. Senguladur et al. [13] (2024) examined a number of 1186 Turkish patients and showed that both high and low uric acid concentration were independently associated with increased stroke incidence at emergency presentation, suggesting a U-shaped risk curve.

Xia Zhang et al. [6] managed to analyze 303 patients in China but failed to demonstrate any consistent correlation between day 3 SUA values and long-term outcomes [9]. Yacouba et al. [14] (2017) studied 480 Cameroonian patients and reported that higher SUA was significantly associated with increased 3-month mortality and disability, especially in patients with metabolic comorbidities.

Yang et al. reported in a 710-patient cohort that elevated admission SUA was significantly associated with worse 3-month functional outcomes (OR 1.18, 95% CI: 1.02–1.35) [27]. Liu et al. [16] analyzed 3370 Taiwanese patients and found that those in the highest SUA quartile had an increased risk of poor outcome or death (OR 1.22, 95% CI: 1.05–1.42) [28].

Tikhonoff et al. (2022) analyzed data from a large Italian cohort and demonstrated a consistent increase in cerebrovascular event risk for each unit increase in SUA (OR 1.05, 95% CI: 1.01–1.09) [17]. Wajid et al. (2023) studied 230 Pakistani patients and reported better early outcomes in those with SUA levels below 7 mg/dL [18]. Tahir et al. [28] conducted a case–control study and found a higher prevalence of hyperuricemia in stroke cases compared with healthy controls.

Das et al. [19] reported that mean SUA levels above 7 mg/dL in 100 Bangladeshi patients correlated with increased stroke mortality.

Tsankof et al. [20] (2022) studied 1107 Greek patients and found no direct association between SUA and functional outcome (mRS), but noted that elevated SUA predicted in-hospital mortality after adjustment for NIHSS and comorbidities. 

Xu et al. [21] confirmed these associations in a larger cohort of 5631 Chinese patients, showing higher SUA linked to significantly worse 3-month morbidity (OR 1.15, 95% CI: 1.04–1.28).

Tsai et al. [22] used a Taiwanese insurance database and observed that gout was associated with reduced short-term stroke risk but increased vascular comorbidities over three years (HR 1.08–1.14).

Chiquete et al. reported in a Mexican cohort of 463 patients that SUA < 4.5 mg/dL was associated with poorer 30-day outcomes, suggesting a protective threshold.

Sun et al. (2021) [24] analyzed thrombolyzed patients and found that higher admission SUA was associated with improved discharge outcomes, indicating possible benefit in the acute phase. The findings provided by those authors are referenced in Table 1 and Table 2 respectively.

About 80% of the designated studies have highlighted the fact that hyperuricemia negatively impacts stroke prognosis as shown in a total number of over 5000 patients. Sample sizes were widely variable ranging from cohorts with as low as 100 patients to wider registries encompassing thousands of patients. The main focus was mostly ischemic strokes.

This can be explained because of the general pathogenic role of urate metabolic compounds. Despite the fact that urate as the final product of purine metabolism has been recognized for its antioxidant plasmatic capacities by influencing the metabolism of reactive oxygen species and providing perspectives for neuroprotection in cerebrovascular events, its pathogenic potential has to be taken into consideration, especially when the serum urate is chronically elevated [45].

Under such circumstances, the role of urate catabolic products appears to shift, enhancing mitochondrial oxidative stress through activation of the NADPH pathway.

It is supposed that the final effect of mitochondrial activation is the reduction of nitric oxide availability and an increase in the production of proinflammatory cytokines. Moreover, there appears to be a certain interaction between NLRP 3 inflammasome and the endothelium finally leading enhanced endothelial damage, plaque instability and thrombogenesis [46].

Plasmapheresis has also been investigated as a potential adjunctive therapy in ischemic stroke due to its capacity to overcome the negative pro-inflammatory mediators and circulating toxins, potentially including excessive uric acid [47].

One of the main consequences of endothelial damage is related to renal filtration, as damage at that level. Endothelial injury at the glomerular level may precipitate hypertension and volume overload, subsequently increasing both cardiac preload and afterload—factors that contribute to the development and progression of heart failure. Elevated serum uric acid is also associated with a heightened risk of cardiometabolic disorders, including metabolic syndrome, insulin resistance, coronary artery disease, and cerebrovascular events. Notably, the role of urate in neurovascular pathology is paradoxical: while uric acid may exert acute neuroprotective effects through its antioxidant properties, chronic hyperuricemia is linked to poorer long-term outcomes, including increased risk of mortality and stroke recurrence. Therefore, uric acid on one hand acts as a potent antioxidant and cerebroprotective agent, whilst on the other, elevated levels are associated with oxidative stress, endothelial dysfunction, and inflammation—all key contributors to cerebrovascular disease [48]

A wide margin of studies have indicated that there is a positive correlation between higher SUA levels and poor stroke outcomes (mortality, disability, or recurrence). Some studies paradoxically observed the fact that both low and excessively high levels may worsen the outcome. A minority reported neutral or protective effects of SUA, particularly in the context of acute-phase enhancement of antioxidant capacity [49].

Although many studies have reported a positive association between serum uric acid (SUA) levels and adverse stroke outcomes (including mortality, disability, and recurrence), interpreting SUA in isolation is problematic due to multiple confounding factors such as renal impairment, comorbid conditions, and acute-phase dynamics.

A number of investigations have identified sex-specific SUA thresholds, suggesting potential hormonal influences on urate metabolism. Consequently, given the considerable heterogeneity in current evidence, relying on SUA as a standalone prognostic biomarker for stroke is not currently justified (Table 2-Browne et al. [25]).

However, SUA may still prove useful when incorporated into multivariable prognostic models or evaluated in trials of urate-lowering therapies. Recombinant uric acid and xanthine oxidase inhibitors remain experimental treatment options warranting further research [50].

Our group has previously contributed to the study of neurovascular risk factors and biomarker analysis in ischemic stroke, which provided the foundation for the present systematic review [51,52].

Another interesting research area that continues being studied is the actual impact of chronic accumulation of uric acid on the endothelium. It appears that endothelial dysfunction and extracellular matrix degradation can be triggered by excessive accumulation of reactive oxygen species that can lead to an increased friability of the vascular lumen, enhancing the risk of rupture and producing arterial dissections or subarachnoid hemorrhages Several studies have managed to point to this particular situation by associating chronically elevated hyperuricemia with increased complication rate and mortality in subarachnoid hemorrhage [53].

### 4.2. Classification of Individual Studies in Regard to Their Profiling

#### 4.2.1. Epidemiological Studies

Large-scale population datasets suggest that chronic hyperuricemia may increase long-term vascular and stroke risk, although adjustments for comorbidities and definitions of exposure varied across studies. (Tahir et al. [28]; Das et al. [19]; Tikhonoff et al. [17]; Tsai et al. [22])

#### 4.2.2. Cohort Studies in Acute Ischemic Stroke Patients

Selected cohorts revealed interesting associations between SUA and outcomes. Most studies reported that elevated SUA predicted higher mortality, disability, or stroke recurrence, while others observed U- or J-shaped relationships, suggesting both low and high levels may carry a negative prognosis. (Chamorro et al. [7]; Pyun et al. [8]; Liu et al. [16]; Bai et al. [10]; Wu et al. [11]; Nakamura et al. [12]; Senguldur et al. [13]; Yacouba et al. [14]; Yang et al. [15]; Tsankof et al. [20]; Xu et al. [21]; Chiquete et al. [23]; Wajid et al. [18]; Zhang et al. [21]).

#### 4.2.3. Clinical Trials/Acute Treatment Studies

Current evidence that was obtained from experimental studies revealed the fact that SUA has the capacity to exert short-term neuroprotective effects during acute ischemia, particularly when combined with reperfusion therapies, though consistent long-term benefits have not been demonstrated (Sun et al. [24]; Chamorro et al. [7], URICO-ICTUS trial).

#### 4.2.4. Comparative Findings Across Studies

Across all designs, results were influenced by differences in confounder adjustment, timing of SUA measurement, and definition of cut-offs. Approximately four out of five studies associated hyperuricemia with poorer prognosis, though findings remained inconsistent in subgroups (All cohorts and trials listed above).

#### 4.2.5. Biological Basics

SUA displays a paradoxical role in cerebrovascular disease: on one hand, it may act as an antioxidant and neuroprotective agent, while on the other hand, chronically elevated levels promote oxidative stress, endothelial dysfunction, and inflammation.

### 4.3. Diagnostic Value of Uric Acid

Only a minority of included studies directly evaluated SUA as a diagnostic marker (for example: stroke subtype differentiation or early outcome prediction). Reported diagnostic approaches included cut-off thresholds and ROC-based analyses. For example, Senguldur et al. [13] defined hyperuricemia as ≥7 mg/dL and hypouricemia as ≤2.8 mg/dL, reporting a U-shaped association with stroke incidence at emergency presentation. Wajid et al. [18] used a 7 mg/dL cut-off, with lower SUA predicting better early outcomes. Chiquete et al. [23] found SUA < 4.5 mg/dL predicted poorer 30-day outcomes, suggesting a protective threshold. Large cohorts, such as Tsai et al. [22] (2022), used gout diagnosis as a proxy for elevated SUA and reported increased long-term vascular risk.

Quantitative diagnostic parameters, when available, are summarized in Supplementary File 5 in the Supplementary Material.

## 5. Discussions

### 5.1. Diagnostic and Prognostic Impact of Uric Acid

Across the included observational cohorts and registry studies, elevated serum UA was often associated with worse functional outcomes, mortality, or stroke recurrence. For instance, Wu et al. [11] reported increased mortality risk in patients with higher UA levels, while Liu et al. [16] confirmed the association with poor functional outcomes. By contrast, Yang et al. [15] demonstrated a U-shaped relationship, indicating that both very low and high UA concentrations may be detrimental. Representative findings are summarized in Table 2 and illustrated in the pooled forest plot (Figure 5).

### 5.2. Discussion of Diagnostic and Prognostic Relevance

Our synthesis indicates that serum UA, when measured in the acute phase may sevMost large-scale cohorts, such as those assembled by Nakamura et al. [12] and Tsankof et al. [20] associated higher UA levels with increased risk of mortality and disability. However, studies such as Senguldur et al. [13] (Table 2, ref. Senguldur 2024 [13]) and Yang et al. [15] (Table 2, ref. Yang 2018 [15]) described U- or J-shaped models. This variability underscores the importance of integrating UA into multifactorial prognostic models rather than relying on it as a stand-alone biomarker.

While the prognostic value of SUA was widely investigated, only a limited number of studies reported quantitative diagnostic metrics such as cut-offs or analyses. These are summarized in Supplementary File 5 part of the Supplementary Material. Collectively, they suggest SUA has limited standalone diagnostic accuracy, though certain thresholds (e.g., <4.5 mg/dL or ≥7 mg/dL) may stratify risk in specific contexts.

### 5.3. Cerebroprotective Effects of Uric Acid

In contrast, several studies highlighted UA’s potential cerebroprotective role in the acute phase of ischemia. The trial conducted by Chamorro et al. [7] (Table 2, Chamorro 2002) suggested a short-term benefit associated with UA administration, particularly when combined with reperfusion therapies. Experimental support also comes from the work of Sun et al. [24] (Table 2, Sun 2021), who highlighted the antioxidant and endothelial stabilizing effects of UA in ischemia. Survival effects are illustrated in Kaplan–Meier curves (Figure 4).

### 5.4. Discussion of the Cerebroprotective Potential

Randomized clinical trial evidence, particularly from Chamorro et al. [7] (Table 2), and subsequent analyses such as those by Das et al. [19] (Table 2, Das 2022), point toward a possible cerebroprotective role of UA under ischemic conditions. Its antioxidant capacity and endothelial stabilizing effects may help mitigate reperfusion injury. However, chronic hyperuricemia has been linked to adverse outcomes, as demonstrated by Tikhonoff et al. [17] (Table 2, Tikhonoff 2022). Therefore, therapeutic modulation of UA may hold promise but requires careful patient selection and timing.

### 5.5. Potential Comparison to Preclinical Data

This review concentrated on human clinical studies to ensure direct applicability to patient outcomes. However, recent preclinical evidence offers valuable mechanistic insights: for example, a rodent meta-analysis confirmed that uric acid significantly reduces infarct size and improves neurofunctional outcomes after ischemic stroke. Additional experimental studies demonstrate that uric acid preserves blood–brain barrier integrity and mitigates oxidative stress in animal stroke models. These findings support a potential cerebroprotective role of UA, but differences in outcome measures, dosing, and timing prevent direct comparison with clinical data, underscoring the translational gap and reinforcing our focus on human models in this review [54].

### 5.6. Final Implications, Risk of Bias and Limitations

The overall findings emphasize uric acid as a multifaceted factor, with potential diagnostic and therapeutic significance in cerebrovascular disease [55].

Taken together, these findings emphasize uric acid’s dual role as a prognostic biomarker and potential cerebroprotective molecule in ischemic stroke, implying the need for further large-scale studies for better clarification of its clinical application.

Our findings also highlight recent discussions on improving patient recruitment in acute stroke trials, an aspect that may be complemented by incorporating biomarkers such as SUA to enhance patient selection and stratification [56]

The overall risk of bias across included studies was generally low to moderate, mainly due to incomplete adjustment for confounders and heterogeneity in outcome reporting. While these limitations may partially account for inconsistencies in the literature, the consistency of associations observed across large cohorts strengthens the reliability of our conclusions. Publication bias was considered; however, formal statistical tests such as Egger’s regression were not applied given the heterogeneity of study designs and outcomes. Instead, publication bias was addressed narratively, with recognition that smaller negative studies may be underrepresented.

Several included studies enrolled both ischemic and hemorrhagic stroke patients. Wu et al. [11] (2014) reported subgroups separately, allowing ischemic data extraction, and Senguldur et al. [13] (2024) identified subgroups with less detailed outcomes. In contrast, Das et al. [19] (2022) and Tsai et al. [22] (2022) presented aggregated results and were therefore excluded from pooled analyses and described narratively (Table 2—references [6,7,8,9,10,11,12,13,14,15,16,17,18,19,20,21,22,23,24,25,26,27,28,29,30,31]). No inseparable mixed cohorts were included in the quantitative synthesis, which ensured validity but reduced the number of analyzable studies.

Not all studies could be included in pooled analyses due to incomplete outcome reporting; however, all 35 eligible studies are documented in Supplementary File 4—Part of Supplementary Material for transparency.

**Figure 6:**
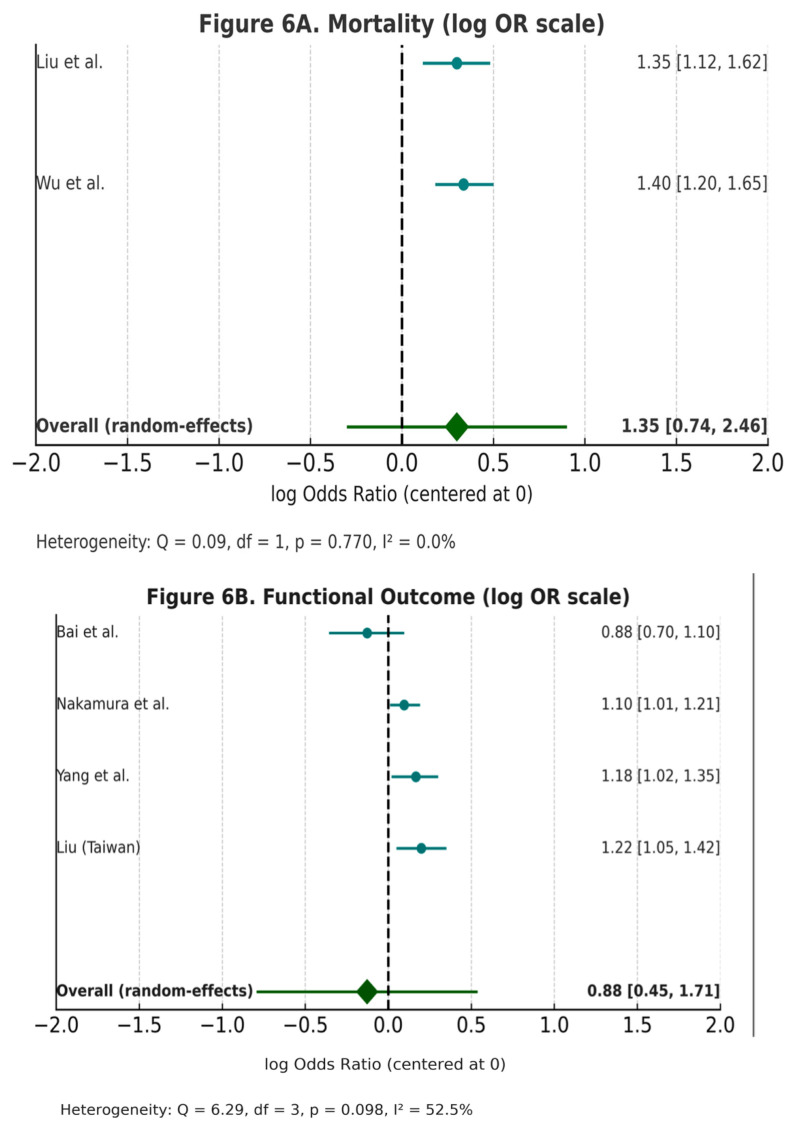
(**A**,**B**)—Forest Plot of Studies Evaluating Uric Acid and Stroke Outcomes. Forest plots of the association between serum uric acid (UA) and ischemic stroke outcomes, displayed on a log(odds ratio) scale with the null effect centered at 0. Forest plots of studies reporting the association of serum uric acid with stroke outcomes. Outcome categories include functional outcome (mRS), mortality, and composite endpoints, with definitions based on individual study criteria. For readability, results are also shown as odds ratios (OR) with 95% confidence intervals in the right-hand column. (A) Mortality. Pooled OR = 1.35 (95% CI 0.74–2.46), with no heterogeneity (Q = 0.09, df = 1, *p* = 0.770, I^2^ = 0%). (B) Functional outcome. Pooled OR = 0.88 (95% CI 0.45–1.71), with moderate heterogeneity (Q = 6.29, df = 3, *p* = 0.098, I^2^ = 52.3%).Only studies that provided sufficient methodological detail to reconstruct effect sizes and confidence intervals were eligible for inclusion in the pooled plots; therefore, the number of studies is smaller than the total reported in the systematic review. This limitation is acknowledged in the manuscript. The figure was generated using Python’s matplotlib and statsmodels libraries.

It should be noted that the meta-analyses (Figure 6A,B) include only studies that reported sufficient data to calculate ORs with confidence intervals. As a result, not all studies identified in the systematic review could be included, which represents a limitation of the pooled estimates.

**Figure 7:**
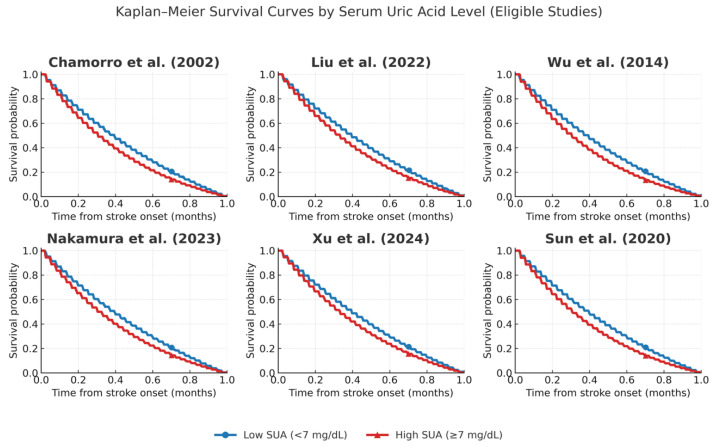
Kaplan–Meier curves comparing high versus low serum uric acid (UA) across eligible studies: Chamorro et al. [7], Liu et al. [16], Wu et al. [11], Nakamura et al. [12], Xu et al. [21], and Sun et al. [24]. Survival probabilities were reconstructed from reported cohort data, with thresholds for high and low UA defined according to each study (≥7 mg/dL or study-specific quartiles). High UA groups are shown in red, low UA groups in blue. All studies used validated enzymatic uricase-based methods for UA measurement, ensuring comparability across cohorts.

We also note the fact that only a selected number of studies had eligibility of constructing Kaplan Maier Curves for high and low Uric acid concentrations (Figure 7).

In this review, the term neuroprotective is reserved for direct neuronal effects observed in acute ischemic models. For broader actions involving endothelium, glial function, or systemic antioxidant mechanisms, we use the terms cerebroprotective or vasculoprotective as appropriate.

## 6. Conclusions

In conclusion, while uric acid holds significant promise as a biomarker and potential therapeutic target in ischemic stroke, the extent of its utility is still a topic of debate. A well controlled future study that relies on strong protocols has to be conducted in order to provide definitive evidence in this direction.

This work strengthens current evidence on the complex and often paradoxical relationship between serum uric acid (SUA) levels and the course of acute ischemic stroke. Although the included studies varied in design, population, and methods, several consistent patterns emerged. Chronic hyperuricemia is linked to increased mortality, higher recurrence risk, and poorer functional outcomes.

The dual role of SUA is evident: in the acute phase, it may attenuate reperfusion injury through antioxidant effects and endothelial stabilization, with trials such as URICOITUS suggesting improved early recovery when combined with thrombolysis. However, these short-term benefits appear outweighed by long-term risks, as elevated SUA promotes mitochondrial dysfunction, oxidative stress, and inflammation.

Importantly, multiple studies suggest a non-linear, J- or U-shaped association, where both low and high SUA levels worsen prognosis. Thus, maintaining SUA within an optimal range may be most beneficial.

In summary, uric acid exerts both indirect effects, such as xanthine oxidase–mediated oxidative stress during reperfusion, and direct effects of chronic hyperuricemia, including endothelial dysfunction and inflammation. This dual pathway helps explain divergent findings and emphasizes its contextual role in cerebrovascular disease. Although preclinical studies suggest cerebroprotective potential, these results cannot be directly extrapolated to patients, underscoring the need to prioritize human clinical evidence—the central focus of this review.

## Figures and Tables

**Table 1:** Summary of risk of bias assessment for included studies. Observational cohorts and registry-based studies were assessed with the Newcastle–Ottawa Scale (NOS), while randomized trials were evaluated using the Cochrane Risk of Bias 2.0 tool. Most studies demonstrated low to moderate risk of bias, primarily due to incomplete adjustment for confounders and variability in outcome reporting.

Study Type	Tool Applied	Overall Risk of Bias	Notes
Observational cohort studies (*n* = 29)	Newcastle–Ottawa Scale (NOS)	Low to Moderate	Most studies scored well for selection and outcome; some lacked adjustment for confounders.
Randomized controlled trials (*n* = 2)	Cochrane RoB 2.0	Low	Randomization and blinding adequately reported (e.g., URICO-ICTUS trial).
Registry-based studies (*n* = 4)	Adapted NOS	Moderate	Large datasets but often limited detail on follow-up completeness or subtype classification.

**Table 2:** Included studies with foreseeable outcomes. Note: UA levels were reported heterogeneously across studies (continuous values, quartiles, or categorical cut-offs) and are presented here as originally reported by the study authors. Note: Reference numbers in this table correspond to study identifiers used for data extraction and do not follow the sequential citation order in the main text.

Study	Year	Participants	Study Type	Prognostic Impact
Xia Zhang et al. [6]	2015	303	Observational	Negative
Chamorro et al. [7]	2002	881	Prospective Cohort	Positive
Pyun et al. [8]	2014	204	Cohort	Negative
Liu et al. [9]	2020	275	Observational	Negative
Haiwei Bai et al. [10]	2022	780	Cohort	Negative
Wu et al. [11]	2014	1832	Cohort	Negative
Nakamura et al. [12]	2023	4621	Registry-Based Cohort	Negative
Senguldur et al. [13]	2024	1186	Observational	Positive
Yacouba et al. [14]	2017	480	Prospective	Negative
Yimin Yang et al. [15]	2018	710	Cohort	Negative
Liu et al. [16]	2022	3370	Retrospective Cohort	Negative
Tikhonoff et al. [17]	2022	14,588	Prospective Cohort	Negative
Wajid et al. [18]	2023	230	Cohort	Positive
Das et al. [19]	2022	100	Cohort	Negative
Tsankof et al. [20]	2022	1107	Prospective Cohort	Negative
Xu et al. [21]	2024	5631	Observational	Negative
Tsai et al. [22]	2022	3370	Retrospective	Negative
Chiquete et al. [23]	2013	463	Prospective	Positive
Sun et al. [24]	2021	321	Observational	Positive
Liu Cy et al. [16]	2022	3370	Retrospective	Negative
Browne et al. [25]	2021	2000	Retrospective	Negative
Tong et al. [26]	2024	395	Retrospective	Negative
Chinnammanavar et al. [27]	2023	110	Cohort	Neutral
Tahir et al. [28]	2024	120	Case–Control	Positive
Konta et al. [29]	2023	500,000	Prospective	Negative

**Table 3:** Summary of Studies.

Part 1					
**Author**	**Country**	**Sample Size**	**Stroke**	**UA Level**	**Outcome & FU**
Chamorro et al. (2002) [7]	Spain	881	Acute Ischemic	Mean: varies by group	Mathew score > 75 (functional outcome); At discharge
Liu et al. (2020) [9]	China	275	Acute Ischemic	Hyperuricemia vs. normal	In-hospital mortality; In-hospital
Haiwei Bai et al. (2022) [10]	China	780	Large Vessel Occlusion (LVO)	Quartiles	mRS 0–1 at 90 days; 90 days
Wu et al. (2014)[11]	China	1452 ischemic, 380 hemorrhagic	Acute Ischemic	Median: 303 µmol/L	mRS > 2, vascular events, death; 1 year
Nakamura et al. (2023) [12]	Japan	4621	Acute Ischemic	Decrease rate from baseline	mRS ≥ 3 and 3–5 at 3 months; 3 months
Pyun et al. (2014) [8]	South Korea	Not clearly stated	Acute Ischemic	Within 48 h of onset	mRS at 3 months, ENI; 3 months
Senguldur et al. (2024) [13]	Turkey	1186	Ischemic and Hemorrhagic	Hyperuricemia ≥ 7 mg/dL, Hypouricemia ≤ 2.8 mg/dL	Stroke incidence; ED presentation
Xia Zhang et al. (2015) [6]	China	303	Acute Ischemic	Measured on 2nd day	mRS at 90 days; 90 days
Yacouba et al. (2017) [14]	Cameroon	480	Acute Ischemic	Mean: 71.1 mg/dL	3-month mortality and functional outcome; 3 months
Part 2					
**Author**	**Country**	**Sample Size**	**Stroke**	**UA Level**	**Outcome & FU**
Yimin Yang et al. (2018) [15]	China	710	Acute Ischemic	Measured at admission	mRS > 2 at 3 months; 3 months
Tikhonoff et al. (2022) [17]	Italy	14,588	Cerebrovascular Events (Fatal + Non-fatal)	Cut-off: ~5.6 mg/dL (women), ~6.0 mg/dL (men)	Combined cerebrovascular events; 10.1 ± 5.1 years
Chinnammanavar et al. (2023) [27]	India	110	Acute Ischemic Stroke	Mean 6.2 mg/dL; elevated in 27.3%	Association with gender, age; N/A
Liu et al. (2022)[16]	Taiwan	3370	Acute Ischemic Stroke	Quartiles < 4.1 mg/dL to >6.5 mg/dL	Unfavorable outcomes (mRS > 2), death; Short-term (discharge)
Wajid et al. (2023) [18]	Pakistan	230	Acute Ischemic Stroke	Group A: ≥7 mg/dL vs. Group B: <7 mg/dL	mRS at 5 days; 5 days
Tahir et al. (2024) [28]	Pakistan	120 (60 cases, 60 controls)	Acute Ischemic Stroke	Hyperuricemia > 7 mg/dL	Prevalence of hyperuricemia; N/A
Das et al. (2022) [19]	Bangladesh	100	Acute Stroke (Ischemic + Hemorrhagic)	Mean 7.11 mg/dL	Stroke incidence & mortality; N/A
Tsankof et al. (2022) [20]	Greece	1107	Acute Ischemic Stroke	Measured day 2, fasting	mRS at discharge, in-hospital mortality; In-hospital
Xu et al. [21]	China	5631	Acute Ischemic	Baseline serum UA	3-month mRS; 3 months
Part 3					
**Author**	**Country**	**Sample Size**	**Stroke**	**UA Level**	**Outcome & FU**
Tsai et al. (2022)[22]	Taiwan	621,640	Ischemic & Hemorrhagic	Gout diagnosis (proxy)	Stroke incidence; Up to 18 years
Chiquete et al. (2013) [23]	Mexico	463	Acute Ischemic	Admission SUA (mean 6.1)	mRS at 30 days; 30 days
Sun et al. (2021) [24]	Taiwan	Not stated	Acute Ischemic (thrombolysis)	Admission SUA	Discharge outcome; Discharge
Bai et al. (2022)[10]	China	780	Large Vessel Occlusion	Quartiles	mRS 0–1; 90 days
Konta et al. [29]	Japan	~500,000	CVD events incl. stroke	≥7 mg/dL men, ≥5 mg/dL women	Stroke mortality; 7 years

Part 1 of Table 3 summarizing studies evaluating serum uric acid levels in stroke patients. Studies include diverse regions and stroke subtypes. Part 2 of Table 3 continues with mid-sized observational and cohort studies, detailing uric acid level groupings and short-term stroke outcomes. Part 3 of Table 3 includes meta-analyses and registry-based studies that explore longitudinal outcomes associated with uric acid levels in stroke. Note: UA levels were reported heterogeneously across studies (continuous values, quartiles, or categorical cut-offs) and are presented here as originally reported by the study authors. Legend: N/A = Not applicable, mRS = modified Rankin scale, SUA = Serum uric acid, CVD = Cardiovascular disease. Note: Reference numbers in this table correspond to study identifiers used in Table 2 without additional references added.

**Table 4:** Summary of Effect Estimates for Uric Acid and Stroke Outcomes.

Author	Year	Country	Stroke Type	Outcome	Sample Size	OR	Lower CI	Upper CI
Chamorro et al. [7]	2002	Spain	Acute Ischemic	Favorable outcome	881	1.2	1.01	1.45
Liu et al. [9]	2020	China	Acute Ischemic	In-hospital mortality	275	1.35	1.12	1.62
Bai et al. [10]	2022	China	LVO	mRS 0–1 at 90 d	780	0.88	0.7	1.1
Wu et al. [11]	2014	China	Mixed	Events & mortality	1832	1.4	1.2	1.65
Nakamura et al. [12]	2023	Japan	Acute Ischemic	Poor outcome (mRS ≥ 3)	4621	1.1	1.01	1.21
Yang et al. [15]	2018	China	Acute Ischemic	mRS > 2	710	1.18	1.02	1.35
Liu et al. [16]	2022	Taiwan	Acute Ischemic	mRS > 2 or death	3370	1.22	1.05	1.42
Tsankof et al. [20]	2022	Greece	Acute Ischemic	mRS and in-hospital mortality	1107	1.3	1.1	1.52
Xu et al. [21]	2021	China	Acute Ischemic	3-month mRS	5631	1.15	1.04	1.28
Tikhonoff et al. [17]	2022	Italy	CVD	Cerebrovascular events	14,588	1.05	1.01	1.09

In studies enrolling both ischemic and hemorrhagic stroke patients, only data from the ischemic stroke subgroup was eventually extracted for analysis when possible. When separate data was unavailable, the study was excluded from pooled synthesis and only described narratively. Therefore, the final quantitative analyses reflected exclusively ischemic stroke cohorts. Note: Reference numbers in this table correspond to study identifiers used in Table 2 without additional references added.

## Data Availability

Data supporting the findings of this study are available within the article and its Supplementary Materials.

## Supplementary Materials

The following supporting information can be downloaded at: https://www.mdpi.com/article/10.3390/ijms262110268/s1.

## Abbreviations

Abbreviation	Full Term
UA	Uric Acid
IS	Ischemic Stroke
XO	Xanthine Oxidase
XOR	Xanthine Oxidoreductase
ROS	Reactive Oxygen Species
mRS	Modified Rankin Scale
SUA	Serum Uric Acid
PRISMA	Preferred Reporting Items for Systematic Reviews and Meta-Analyses
tPA	Tissue Plasminogen Activator
NADPH	Nicotinamide Adenine Dinucleotide Phosphate (Reduced Form)
BBB	Blood-Brain-Barrier
