# Supplementary material for: Uric Acid in Cerebral Ischemia: A Systematic Review of Its Biomarker Value and Role in Neuroprotection
Source: Int J Mol Sci. 2025 Oct 22;26(21):10268. doi: 10.3390/ijms262110268 (PMC12610115; doi:10.3390/ijms262110268)
Supplement: Supplementary file 1 [file ijms-26-10268-s001.zip › Supplementary File 1-Database search strategies.pdf]

Supplementary Material – Detailed Search Strategies

This supplementary file provides the detailed search algorithms applied across the selected databases (PubMed, Cochrane Library, and Google Scholar). Four alternative search strings were tested for each database to ensure completeness, sensitivity, and reproducibility.

PubMed

1. ("Uric Acid"[Mesh] OR "uric acid"[tiab] OR urate[tiab]) AND ("Stroke"[Mesh] OR "ischemic stroke"[tiab] OR "cerebral infarction"[tiab]) AND ("Prognosis"[Mesh] OR prognosis[tiab] OR outcome*[tiab])

2. ("uric acid"[tiab] OR urate[tiab]) AND ("stroke"[tiab] OR "brain infarction"[tiab]) AND (mortality[tiab] OR recovery[tiab] OR disability[tiab])

3. ("uric acid"[tiab]) AND ("stroke"[tiab]) AND ("functional outcome"[tiab] OR "modified Rankin Scale"[tiab])

4. ("uric acid"[tiab]) AND ("ischemic stroke"[tiab]) AND ("NIHSS"[tiab] OR "neurological outcome"[tiab])

Cochrane Library

1. ("uric acid" OR urate) in Title Abstract Keyword AND ("ischemic stroke" OR "cerebral infarction") AND (prognosis OR outcome*)

2. ("uric acid" OR urate) AND (stroke OR "brain infarction") AND (mortality OR survival)

3. ("uric acid" OR urate) AND (stroke) AND ("functional recovery" OR disability)

4. ("uric acid" OR urate) AND (ischemia OR "ischemic stroke") AND (prognosis OR risk)

Google Scholar

1. allintitle: "uric acid" "ischemic stroke" prognosis OR outcome

2. "uric acid" AND "stroke" AND (prognosis OR mortality OR outcome)

3. "uric acid" AND "ischemic stroke" AND "functional recovery"

4. "uric acid" AND "cerebral infarction" AND (survival OR disability)
