# Supplementary material for: Uric Acid in Cerebral Ischemia: A Systematic Review of Its Biomarker Value and Role in Neuroprotection
Source: Int J Mol Sci. 2025 Oct 22;26(21):10268. doi: 10.3390/ijms262110268 (PMC12610115; doi:10.3390/ijms262110268)
Supplement: Supplementary file 1 [file ijms-26-10268-s001.zip › Supplementary File 2-Risk of Bias Assessment.pdf]

Supplementary Material – Risk of Bias Assessment

This supplementary file provides the detailed risk of bias assessments for all 35 included studies. Observational cohort and registry-based studies were assessed using the Newcastle–Ottawa Scale (NOS), while randomized controlled trials were assessed with the Cochrane Risk of Bias 2.0 tool. Scores and domain-level judgments are presented below.

Author (Year)	Study Type	Tool Applied	Overall Risk of Bias	Comments
Chamorro et al. (2002)	Prospective cohort	NOS	Low	Adequate sample size; reliable outcomes; limited confounder adjustment.
Liu et al. (2021)	Observational cohort	NOS	Moderate	Good mortality assessment; residual confounding possible.
Haiwei Bai et al. (2022)	Cohort	NOS	Moderate	Quartile grouping clear; outcome ascertainment adequate; confounding adjustments limited.
Wu et al. (2014)	Cohort	NOS	Moderate	Large cohort; outcome robust; incomplete confounder adjustment.
Nakamura et al. (2023)	Registry-based cohort	NOS (adapted)	Moderate	Large dataset; incomplete reporting of follow-up.

Pyun et al. (2014)	Cohort	NOS	Moderate	Reasonable design; insufficient details on covariate control.
Senguldur et al. (2024)	Observational cohort	NOS	Moderate	Adequate sample; U-shaped risk described; some outcome measurement issues.
Xia Zhang et al. (2015)	Observational	NOS	Moderate	Limited sample size; outcome assessment adequate.
Yacouba et al. (2017)	Prospective cohort	NOS	Moderate	Outcome measured at 3 months; sample size smaller; confounder adjustment missing.
Yimin Yang et al. (2018)	Cohort	NOS	Low	Clear outcome definition; adequate control for covariates.
Tikhonoff et al. (2022)	Prospective cohort	NOS	Low	Large sample; long follow-up; well adjusted for confounders.
Pavan Kumar et al. (2023)	Cohort	NOS	Moderate	Small sample size; limited adjustment for confounders.
Liu et al.	Retrospective cohort	NOS	Moderate	Large cohort;

(2022)				outcomes valid; adjustment limited.
Wajid et al. (2023)	Cohort	NOS	Moderate	Short follow-up; adequate outcome ascertainment.
Tahir et al. (2024)	Case-control	NOS (adapted)	Moderate	Case-control design; potential recall bias.
Das et al. (2022)	Cohort	NOS	Moderate	Small sample; outcomes adequately reported.
Veneti et al. (2022)	Prospective cohort	NOS	Moderate	Good outcome definition; incomplete confounding adjustment.
Xu et al. (2021)	Observational cohort	NOS	Moderate	Large dataset; robust outcomes; some risk of selection bias.
Tsai et al. (2022)	Registry-based cohort	NOS (adapted)	Moderate	National dataset; proxy measure (gout); misclassification possible.
Chiquete et al. (2013)	Prospective cohort	NOS	Low	Clear SUA definition; outcome robust; adequate follow-up.
Sun et al. (2021)	Observational	NOS	Moderate	Discharge outcomes measured;

				sample size limited.
Bai et al. (2022)	Cohort	NOS	Moderate	Quartiles defined; limited confounder adjustment.
Yamato et al. (Japan registry)	Registry-based cohort	NOS (adapted)	Moderate	Large registry; SUA categories; limited covariates.
URICO-ICTUS Trial	Randomized controlled trial	RoB 2.0	Low	Adequate randomization; blinding and outcome measurement robust.
Tong et al. (2024)	Observational (NHANES)	NOS	Low	National dataset; robust adjustment; adequate reporting.
Zhong et al. (2024)	Cohort + meta-analysis	NOS	Moderate	Well conducted cohort; some heterogeneity in pooled data.
Ndrepepa (2025)	Narrative/epidemiological cohort	NOS	Moderate	Adequate reporting; residual confounding possible.
Otani et al. (2023)	Narrative review	Not applicable	-	Narrative data; not scored.
Muir et al. (2008)	Cohort	NOS	Low	Well reported outcomes; adequate adjustments.

Browne et al. (2021)	Cohort	NOS	Low	Large dataset; robust methodology.
Liu CY et al. (2022)	Cohort	NOS	Moderate	Adequate outcomes; J-shaped risk identified; residual confounding.
Roman-Filip et al. (2024)	Case series	Not applicable	-	Small descriptive case series; not scored.
Roman-Filip et al. (2023)	Narrative review	Not applicable	-	Not scored.
Wang et al. (2025)	Cohort	NOS	Moderate	Moderate sample; outcome ascertainment adequate.
