# Supplementary material for: Uric Acid in Cerebral Ischemia: A Systematic Review of Its Biomarker Value and Role in Neuroprotection
Source: Int J Mol Sci. 2025 Oct 22;26(21):10268. doi: 10.3390/ijms262110268 (PMC12610115; doi:10.3390/ijms262110268)
Supplement: Supplementary file 1 [file ijms-26-10268-s001.zip › Supplementary File 3 Effect Estimates on Forrest plots.pdf]

Supplementary File 3. Summary of Effect Estimates

This supplementary file provides expanded data underlying the summary of outcomes and the construction of forest plots presented in the main manuscript. Only studies with sufficient quantitative data (odds ratios [OR], hazard ratios [HR], confidence intervals [CI], or event rates) are included.

Author (Year)	Country / Population	N	Outcome	Effect Estimate (95% CI)	Impact Direction
Chamorro (2014)	Spain, ischemic stroke	881	mRS at discharge	OR 1.20 (1.01–1.45)	Protective
Liu (2019)	China, ischemic stroke	275	In-hospital mortality	OR 1.35 (1.12–1.62)	Harmful
Bai (2020)	China, large vessel occlusion	780	3-month mRS	OR 0.88 (0.70–1.10)	Neutral/Non-sig.
Wu (2020)	China, ischemic + hemorrhagic	1832	Vascular events, mortality	OR 1.40 (1.20–1.65)	Harmful
Nakamura (2023)	Japan, ischemic stroke	4621	Functional outcome	OR 1.10 (1.01–1.21)	Harmful
Yang (2020)	China, ischemic stroke	710	3-month mRS	OR 1.18 (1.02–1.35)	Harmful
Liu (2021)	Taiwan, ischemic stroke	3370	Mortality / poor outcome	OR 1.22 (1.05–1.42)	Harmful
Xu (2022)	China, ischemic stroke	5631	3-month morbidity	OR 1.15 (1.04–1.28)	Harmful
Chiquette (2021)	Mexico, ischemic stroke	463	30-day outcome	Low UA <4.5 mg/dL linked to poorer outcome	Protective threshold
Sun (2021)	China, thrombolyzed	Not reported	Discharge outcome	Higher UA = improved outcomes	Protective
