# Supplementary material for: Uric Acid in Cerebral Ischemia: A Systematic Review of Its Biomarker Value and Role in Neuroprotection
Source: Int J Mol Sci. 2025 Oct 22;26(21):10268. doi: 10.3390/ijms262110268 (PMC12610115; doi:10.3390/ijms262110268)
Supplement: Supplementary file 1 [file ijms-26-10268-s001.zip › Supplementary File 4-All included studies.pdf]

Supplement 4 – Table of All 35 Included and Excluded Studies

Author (Year)	Country	Participants (n)	Study Type	Prognostic Impact of SUA	Notes / Reason not in main tables
Chamorro et al. (2016, URICO-ICTUS)	Spain	881	RCT / cohort	Positive – UA during reperfusion improved early outcomes	Included in Table 2
Bai et al. (2022)	China	780	Prospective EVT cohort	Negative – Higher SUA predicted worse outcome (NS)	Included in Table 2
Wu et al. (2014)	China	1832	Registry (AIS + ICH)	Negative – SUA associated with vascular events and mortality	Included in Table 2
Nakamura et al. (2023)	Japan	4621	Registry	Negative – Higher SUA linked to poor functional outcome	Included in Table 2
Pyun et al. (2014)	Korea	450	Cohort	Neutral – No significant prognostic association	Included in Table 2
Sengüldür et al. (2024)	Turkey	1186	Emergency dept. cohort	U-shaped – Both high and low SUA linked to stroke risk	Included in Table 2
Xia Zhang et al. (2017)	China	303	Cohort	Neutral – No consistent correlation with outcomes	Included in Table 2

Yacouba / Mapoure et al. (2017)	Cameroon	480	Cohort	Negative – Higher SUA linked to 3-month mortality	Included in Table 2
Yang et al. (2018)	China	710	Cohort	Negative – Higher SUA predicted worse functional outcome	Included in Table 2
Tikhonoff et al. (2022)	Italy	>5000	Population-based cohort	Negative – Each SUA unit increased stroke risk	Included in Table 2
Pavan Kumar et al. (2023)	India	200+	Cohort	Negative – Hyperuricemia linked to poor outcomes	Included in Table 2
Liu et al. (2021)	China	275	Cohort	Negative – SUA associated with in-hospital mortality	Included in Table 2
Liu et al. (2022)	Taiwan	3370	Registry	Negative – High SUA quartile had worse outcomes	Included in Table 2
Wajid et al. (2023)	Pakistan	230	Cohort	Negative – Lower SUA linked to better outcomes	Included in Table 2
Tahir et al. (2020)	Pakistan	100	Case–control	Negative – Higher SUA in stroke cases vs controls	Included in Table 2
Das et al.	Banglades	100	Cohort	Negative – SUA >7 mg/dL	Included in

(2022)	h			associated with mortality	Table 2
Veneti et al. (2022)	Greece	1107	Cohort	Negative – SUA predicted in-hospital mortality, not mRS	Included in Table 2
Xu et al. (2021)	China	5631	Registry	Negative – Higher SUA predicted worse 3-month morbidity	Included in Table 2
Chiquete et al. (2013, PREMIER)	Mexico	463	Cohort	Positive – Low SUA (<4.5 mg/dL) associated with poor outcomes	Included in Table 2
Sun et al. (2021)	China	120	Cohort (thrombolyzed)	Positive – Higher SUA improved outcomes after reperfusion	Included in Table 2
Tsai et al. (2022)	Taiwan	3370	Insurance database	Mixed – Short-term ↓ risk, long-term ↑ morbidity	Included in Table 2
Zhong et al. (2024)	China	5631	Cohort + MR	Negative – Higher SUA worsened outcomes; MR supported causality	Included in Table 2
Tong et al. (2024)	USA	395 stroke pts	Population dataset	Negative – SUA predicted higher mortality	Included in Table 2
Browne et	Ireland	2000+	Population-	Negative – High SUA	Included in

al. (2021)			based	thresholds linked to mortality	Table 2
Liu CY et al. (2022)	Taiwan	3370	Registry	Negative – J-shaped association (low & high SUA harmful)	Added from Table 3
Yamato et al. (2023)	Japan	500,000+	Nationwide cohort	Negative – Elevated SUA increased stroke mortality	Added from Table 3
Soren et al. (2018)	India	120	Cohort	Negative – Higher SUA linked to worse outcomes	Excluded (small, low detail)
Peng et al. (2019)	China	340	Cohort	Negative – Higher SUA predicted poor 3-month mRS	Excluded (overlap with larger Chinese studies)
Xu et al. (2019)	China	275	Cohort	Negative – Hyperuricemia associated with in-hospital mortality	Excluded (similar to Liu 2021, redundancy)
Amaro et al. (2016)	Spain	300	RCT substudy	Positive – UA therapy + reperfusion improved outcomes	Excluded (represented by URICO-ICTUS main trial)
Yimin Yang et al. (2019)	China	710	Cohort	Negative – Admission SUA correlated with poor outcome	Excluded (similar to Yang 2018, overlap)
Zhang P et al.	China	1001	IVT cohort	Mixed – Inverted U-shaped	Excluded (narrative, overlapping

(2024)				association with prognosis	design)
Muir et al. (2008)	UK	200+	Cohort (allopurinol)	Exploratory – SUA modification with therapy	Excluded (older, therapy-focused, not direct prognostic cohort)
Additional small single-center cohorts (2000–2010)	Various	<200	Cohorts	Mixed/negative	Excluded (small, low methodological detail, redundancy)
