# Supplementary material for: Uric Acid in Cerebral Ischemia: A Systematic Review of Its Biomarker Value and Role in Neuroprotection
Source: Int J Mol Sci. 2025 Oct 22;26(21):10268. doi: 10.3390/ijms262110268 (PMC12610115; doi:10.3390/ijms262110268)
Supplement: Supplementary file 1 [file ijms-26-10268-s001.zip › Supplementary File 6-PROSPERO registration protocol.pdf]

# Association Between Uric Acid Levels and Clinical Outcomes in Acute Ischemic Stroke: A Systematic Review

*Iulian Roman, Andone Sebastian*

### Citation

Iulian Roman, Andone Sebastian. Association Between Uric Acid Levels and Clinical Outcomes in Acute Ischemic Stroke: A Systematic Review. Not yet published.

PREVIEW

## REVIEW TITLE AND BASIC DETAILS

### Review title

Association Between Uric Acid Levels and Clinical Outcomes in Acute Ischemic Stroke: A Systematic Review

### Condition or domain being studied

Stroke

### Rationale for the review

The purpose of this systematic review aims to synthesize current evidence regarding the association between uric acid levels, purine metabolism pathways, and clinical outcomes in ischemic stroke patients, and to potentially identify key mechanisms and evaluate the potential for new therapies in acute stroke care.

### Review objectives

The purpose of this systematic review aims to synthesize current evidence regarding the association between uric acid levels, purine metabolism pathways, and clinical outcomes in ischemic stroke patients, and to potentially identify key mechanisms and evaluate the potential for new therapies in acute stroke care.

### Keywords

Uric acid; Ischemic stroke; Biomarker

### Country

Romania

## ELIGIBILITY CRITERIA

### Population

*Included*

Adults (≥18 years) with acute ischemic stroke

### Intervention(s) or exposure(s)

*Included*

Serum uric acid levels measured on admission or within 72 hours

### Comparator(s) or control(s)

This review does not have any comparators

### Study design

Only randomized study types will be included.

*Included*

Observational cohort studies (prospective or retrospective), randomized controlled trials, or meta-analyses

### Context

Ischemic stroke is a major cause of disability and death worldwide. Uric acid has emerged as a molecule of interest due to its dual role: antioxidant effects that may offer neuroprotection and pro-oxidant properties associated with vascular damage. While some studies suggest that higher uric acid levels correlate with better outcomes, others link

hyperuricemia to stroke recurrence and mortality. Clinical trials like URICO-ICTUS have explored its therapeutic use with mixed results. This review aims to clarify the prognostic value and potential therapeutic implications of uric acid in acute ischemic stroke.

## SIMILAR REVIEWS

### Check for similar records already in PROSPERO

We did not search PROSPERO for additional records because PROSPERO is a registry of review protocols, not a source of primary studies or published data. Our objective was to identify peer-reviewed evidence suitable for synthesis; therefore, consulting PROSPERO beyond protocol registration would not have yielded eligible studies and was not methodologically necessary.

## TIMELINE OF THE REVIEW

### Date of first submission to PROSPERO

This record has not been submitted.

### Review timeline

Start date: 11 April 2025. End date: 28 July 2025.

### Date of registration in PROSPERO

This record has not been published.

## AVAILABILITY OF FULL PROTOCOL

### Availability of full protocol

A full protocol has been written and uploaded to PROSPERO. The protocol will be made available after the review is completed.

## SEARCHING AND SCREENING

### Search for unpublished studies

Only published studies will be sought.

### Main bibliographic databases that will be searched

The main databases to be searched are *Embase.com*, *MEDLINE* and *PubMed*.

### Search language restrictions

There are no language restrictions.

### Search date restrictions

There are no search date restrictions.

### Other methods of identifying studies

Other studies will be identified by: *reference list checking*.

### Link to search strategy

A full search strategy has been uploaded to PROSPERO. The PDF may be accessed through this link https://www.crd.york.ac.uk/PROSPEROFILES/b534cd230507a32cebe1dd0f84e32ec0.pdf.

### Selection process

Studies will be screened independently by at least two people (or person/machine combination) with a process to resolve differences.

## DATA COLLECTION PROCESS

### Data extraction from published articles and reports

Data will be extracted independently by at least two people (or person/machine combination) with a process to resolve differences.

Authors will not be contacted for further information.

### Study risk of bias or quality assessment

Data will be assessed independently by at least two people (or person/machine combination) with a process to resolve differences.

Additional information will **not** be sought from study investigators if required information is unclear or unavailable in the study publications/reports.

### Reporting bias assessment

Risk of bias due to missing results will not be assessed

### Certainty assessment

Certainty of findings will not be assessed

## OUTCOMES TO BE ANALYSED

### Main outcomes

The main outcome of this review will be to determine the association between serum uric acid levels and functional outcomes in patients with acute ischemic stroke, including mortality, neurological improvement, and stroke recurrence, and to evaluate the therapeutic efficacy of uric acid administration in conjunction with standard reperfusion therapies.

### Additional outcomes

An additional outcome will be the assessment of adverse events associated with uric acid administration, including symptomatic intracranial hemorrhage, renal complications, and cardiovascular events, as well as subgroup analyses based on sex, glycemic status, baseline uric acid levels, and reperfusion method (e.g., thrombolysis vs. mechanical thrombectomy).

## PLANNED DATA SYNTHESIS

### Strategy for data synthesis

No formal data synthesis is planned - data will be described but not combined.

## CURRENT REVIEW STAGE

### Stage of the review at this submission

| Review stage | Started | Completed |
|---|---|---|
| Pilot work | ✓ | ✓ |
| Formal searching/study identification | ✓ | ✓ |
| Screening search results against inclusion criteria | ✓ | ✓ |
| Data extraction or receipt of IPD | ✓ | ✓ |
| Risk of bias/quality assessment | ✓ | ✓ |
| Data synthesis | ✓ | ✓ |

### Review status

The review is completed.

### Publication of review results

Results of the review will be published in English.

## REVIEW AFFILIATION, FUNDING AND PEER REVIEW

### Review team members

**Dr Iulian Roman** (review guarantor and contact) UMFST Targu Mures Romania. Romania.

No conflict of interest decision selected yet.

**Dr Andone Sebastian**. UMFST George Emil Palade Targu Mures. Romania.

No conflict of interest declared.

### Named contact

**Dr Iulian Roman** (iulian_roman2009@yahoo.com). UMFST Targu Mures Romania. Romania.

### Review affiliation

Doctoral School, George Emil Palade University of Medicine Pharmacy and Sciences Targu Mures Romania

### Funding source

Review has no specific/external funding but is supported by guarantor/review team (non-commercial) institutions.

### Peer review

There has been no peer review of this planned review.

## ADDITIONAL INFORMATION

### Review conflict of interest

Declared individual interests are recorded under team member details.. No additional interests are recorded for this review.

### Medical Subject Headings

Brain Ischemia; Humans; Ischemic Stroke; Metabolic Networks and Pathways; Stroke; Uric Acid

### PROSPERO version history

No preview available

### Disclaimer

The content of this record displays the information provided by the review team. PROSPERO does not peer review registration records or endorse their content.

PROSPERO accepts and posts the information provided in good faith; responsibility for record content rests with the review team. The guarantor for this record has affirmed that the information provided is truthful and that they understand that deliberate provision of inaccurate information may be construed as scientific misconduct.

PROSPERO does not accept any liability for the content provided in this record or for its use. Readers use the information provided in this record at their own risk.

Any enquiries about the record should be referred to the named review contact
