# Supplementary material for: Uric Acid in Cerebral Ischemia: A Systematic Review of Its Biomarker Value and Role in Neuroprotection
Source: Int J Mol Sci. 2025 Oct 22;26(21):10268. doi: 10.3390/ijms262110268 (PMC12610115; doi:10.3390/ijms262110268)
Supplement: Supplementary file 1 [file ijms-26-10268-s001.zip › Supplementary_File_5_Quantitative_Diagnostic_Parameters.pdf]

Supplementary File 5. Quantitative diagnostic parameters of serum uric acid (SUA) in ischemic stroke.

Author (Year)	Country	Diagnostic Aim	Cut-off / Definition	Sensitivity / Specificity	AUC / Key Findings
Senguldur et al. (2024) [25]	Turkey	Stroke incidence at emergency presentation	Hyperuricemia ≥7 mg/dL, Hypouricemia ≤2.8 mg/dL	Not reported	U-shaped association; both low and high SUA increased stroke risk
Wajid et al. (2023) [30]	Pakistan	Early outcome prediction (mRS at 5 days)	Hyperuricemia ≥7 mg/dL	Not reported	SUA <7 mg/dL associated with better short-term outcomes
Chiquete et al. (2013) [35]	Mexico	Poor 30-day outcome after ischemic stroke	SUA <4.5 mg/dL	Not reported	Low SUA predicted worse functional outcome
Tsai et al. (2022) [34]	Taiwan	Long-term vascular risk (proxy: gout diagnosis)	ICD-coded gout diagnosis	Not reported	Gout associated with increased long-term stroke risk (HR 1.08–1.14)
